# Association of Herpes Virus Type 1, Cytomegalo Virus and Epstein–Barr Virus to the Pathogenesis of Peri-Implantitis: A Cross-Sectional Study

**DOI:** 10.3390/dj13110492

**Published:** 2025-10-25

**Authors:** Ioana Suciu, Simona Ruta, George Suciu

**Affiliations:** 1 Romanian Academy of Science, ‘Stefan S. Nicolau’ Institute of Virology, 030304 Bucharest, Romania; simona.ruta@umfcd.ro; 2 Clinical Department, Carol Davila University of Medicine and Pharmacy, 020021 Bucharest, Romania; 3 BEIA, R&D Department, 041386 Bucharest, Romania; george@beia.eu

**Keywords:** peri-implantitis, herpesvirus, viral–bacterial interaction, bone loss

## Abstract

**Background/Objectives**: This study explores the potential relationship between herpesvirus infections and the severity and progression of peri-implantitis. A secondary goal is to investigate whether a virus–bacteria interaction may contribute to differences in bone loss patterns between periodontitis and peri-implantitis. **Methods**: Biological samples, including blood, saliva, and peri-implant crevicular fluid, were collected for viral detection. Blood samples were processed at Queen Mary Laboratory in Bucharest, Romania, while saliva and peri-implant crevicular fluid samples were analyzed at the laboratory of ADD Laboral in Malden, the Netherlands. Sterile paper points were used to collect peri-implant crevicular fluid from the deepest peri-implant sites in 43 patients. A nearby tooth was sampled when present, with edentulous patients being the exception. Several clinical parameters were also considered, including implant and dentition status, smoking, gender, implant location, duration of functional loading, periodontal pocket depth (PPD), bleeding on probing (BoP), suppuration (SUP), and periodontal history. **Results**: Epstein–Barr virus (EBV) was detected in 30.2% of cases, Herpes virus (HSV) in 7.0%, and Cytomegalo virus (CMV) in 0%. EBV showed a moderate inverse correlation with probing depth (r = −0.48) in non-smokers with periodontal disease. Viral detection was highest on lingual and mesial surfaces. Peri-implantitis cases exhibited significantly deeper PPD, higher BoP (96.15%), and suppuration (96.15%) compared to healthy implants or teeth. **Conclusions**: An association was observed between the presence of Herpes viruses and increased peri-implantitis severity, suggesting a potential contributory role of viral pathogens in disease progression.

## 1. Introduction

Despite their high success rates, dental implants remain susceptible to complications that may ultimately lead to failure, the underlying causes of which continue to be the subject of numerous scientific research papers [1,2,3,4,5,6,7,8].

Peri-implant diseases are increasingly recognized as significant complications associated with dental implants, potentially compromising long-term treatment outcomes [9,10,11,12,13,14,15,16]. Peri-implant infections are multifactorial pathological conditions characterized by inflammation of the peri-implant mucosa, with or without progressive loss of supporting alveolar bone, beyond the initial bone remodeling, corresponding to peri-implantitis and mucositis, respectively [17,18]. While peri-implant mucositis is confined to the soft tissues, peri-implantitis involves a progressive, and often non-linear, loss of peri-implant bone [19]. Clinically, peri-implantitis may be asymptomatic in early stages or present with signs including mucosal erythema, edema, increased probing depth (PPD), bleeding on probing (BoP), suppuration (SUP), and radiographically detectable bone loss (BL).

The early diagnosis of peri-implantitis is of paramount importance, as it allows for intervention prior to the establishment of an active disease state. This is particularly critical given the current lack of a universally validated, effective, and predictable treatment protocol for peri-implantitis. However, establishing a diagnosis remains challenging due to the variability in diagnostic criteria proposed across studies and among different authors. According to the most recent ITI Consensus Report, BoP, while commonly used, is not a reliable standalone indicator for peri-implantitis, as its presence does not consistently correlate with disease [20]. Although peri-implant probing is useful for monitoring changes in probing depth, it does not provide sufficient information regarding the extent or pattern of BL over time in the absence of radiographic assessment [21,22].

The revised definition of peri-implantitis proposed by Renvert et al. is grounded in the simultaneous presence of clinical signs of peri-implant inflammation and radiographic evidence of bone loss occurring after initial healing [23,24,25,26,27]. However, the assessment of peri-implant bone levels via radiographic imaging is subject to several limitations. Specifically, conventional periapical and panoramic radiographs provide a two-dimensional representation, permitting evaluation of bone loss only at the mesial and distal aspects of the implant, thereby potentially underestimating buccal and lingual bone changes [28,29,30,31,32,33,34]. As a result, reliance on these imaging modalities may lead to underdiagnosis or delayed recognition of peri-implantitis [35,36,37].

Bacterial biofilms play a central role in the pathogenesis of peri-implantitis, serving as the primary etiological factor initiating the inflammatory cascade in peri-implant tissues [38,39,40]. The unique surface characteristics of dental implants, particularly their micro- and macro-topography, facilitate early microbial adhesion and biofilm maturation [41]. Once established, these complex polymicrobial communities exhibit increased resistance to host immune defenses and antimicrobial therapies, thereby promoting a chronic inflammatory environment conducive to soft tissue degradation and progressive bone loss. The persistence and virulence of peri-implant biofilms underscore the critical importance of mechanical debridement and biofilm disruption in both the prevention and management of peri-implant diseases [42].

Human herpesviruses—particularly Herpes Simplex Virus types 1 and 2 (HSV-1, HSV-2), Cytomegalovirus (CMV), and Epstein–Barr Virus (EBV)—have been more frequently identified in periodontal lesions than in the peri-implant inflammatory ones. These viruses possess the ability to establish latency within host cells and can undergo reactivation in response to systemic or local immunological stressors. Upon reactivation, they may alter host immune function and contribute to the amplification of tissue destructive processes. Although their presence has been consistently associated with sites of active periodontitis, the extent to which herpesviruses are implicated in the onset and progression of peri-implantitis remains inadequately characterized [43]. The aim of the study is to evaluate the association of HSV, CMV, and EBV with peri-implantitis and assess their interaction with clinical and bacterial parameters.

## 2. Materials and Methods

### 2.1. Study Design and Patient Recruitment

The cross-sectional study was carried out in compliance with the ethical principles outlined in the Declaration of Helsinki, as set forth by the World Medical Association. Participants were enrolled from the private medical practice owned by the main author. The STROBE guidelines were followed.

Eligibility criteria for inclusion in the study required participants to be over 18 years of age, in general good health, and to have at least one functional dental implant. Exclusion criteria included the use of systemic antibiotics within the last 6 months, any chronic medical conditions, pregnancy or lactation, implant mobility, a time of functional implant loading of less than 1 year, and less than one year of periodontal or peri-implant surgery prior to sampling. Partially edentulous patients were also excluded. Prior to participation, all individuals were provided with detailed information regarding the study’s objectives, benefits, and they gave written informed consent. Each participant completed the following four documents:

- Medical History and Health Status Questionnaire;
- Patient Informed Consent for the Collection, Storage, and Use of Biological Samples for Medical Testing Purposes;
- Patient Consent for Participation in the Clinical Study;
- Patient Consent for the Processing of Personal Data (in accordance with the General Data Protection Regulation—GDPR).

### 2.2. Clinical Examination

The following parameters were recorded: age, gender, dentition status, smoking habits, implant location, duration of functional loading, probing PPD, presence of BoP, suppuration (SUP), oral hygiene, and smoking habits. Clinical parameters were assessed at one site per implant or tooth (buccal, distal, mesial, or lingual). Intraoral periapical radiographs were performed at a radiology center, and peri-implant bone levels were evaluated.

The diagnosis of implant health and disease was made based on the criteria outlined in the 2017 World Workshop on the Classification of Periodontal and Peri-Implant Diseases and Conditions [20]. PI was diagnosed based on clinical signs of inflammation, a probing PPD of ≥6 mm, and radiographic bone loss of ≥3 mm from the implant shoulder [20]. For the purpose of data analysis, pockets with a PPD < 5 mm were categorized as shallow, while those with a PPD ≥ 5 mm were classified as deep. If a subject presented with both healthy implants and implants affected by peri-implantitis, classification was based on the most severe diagnosis; therefore, the individual was assigned to the peri-implantitis group.

### 2.3. Collection of Peri-Implant Biofilm Samples and Subsequent DNA Extraction

For each patient, a specific kit was utilized. Each kit contained four color-coded 1.5 mL screw cap micro tubes (Sarstedt, Germany) (blue, yellow, orange, and black), sterile paper points, (# 50, Spident, Incheon, Republic of Korea), a pipette and a saliva collection tube (Salivette Cortisol, Sarstedt, Germany). Each tube was designated for either the implant or a natural tooth. The implant’s health status was categorized as either a healthy implant (HI) or PI.

#### Microbiological Sampling Method

Patients were instructed to refrain from eating, drinking, or brushing their teeth for at least 30 min prior to sample collection. Prior to microbiological sampling, the selected sites were carefully isolated using cotton rolls to minimize the risk of salivary contamination. Following the careful removal of supragingival plaque using a curette or gentle air stream, subgingival plaque samples were collected using sterile paper points. Throughout the entire procedure, particular attention was given to avoid inducing any sulcular bleeding.

Crevicular fluid samples were obtained from one predetermined site (buccal, lingual/oral, mesial, and distal) using five sterile paper points per collection, each of which was held in place for 15 s. All five paper points were then placed in the respective color-coded tube. 

Patients were sampled according to the following protocol: a maximum of four sites per patient were included, with at least one site corresponding to a neighboring natural tooth. In cases of peri-implantitis, the adjacent tooth to the affected implant was specifically selected for sampling.

If a participant presented multiple implants exhibiting the same peri-implant disease diagnosis, biofilm samples were collected from two to a maximum of three distinct implant sites per individual. In addition, one sample was mandatorily collected from a neighboring natural tooth.

Saliva samples were collected using a pipette and transferred into a designated collection tube. Additionally, a certified phlebotomy nurse obtained one tube of blood from each participant.

### 2.4. DNA Extraction and qPCR Analysis

After collecting the samples, they were sent by post to the laboratory. Once the samples were received, DNA was extracted from both the saliva samples and the crevicular fluid.

The salivettes containing saliva were centrifuged. Fifty microliters of saliva were transferred to a clean tube, to which 150 µL of InstaGene™ Matrix (Bio-Rad, Hercules, CA, USA) was added. Alternatively, 150 µL of InstaGene™ Matrix was added directly into the Eppendorf tube containing the paper points. The InstaGene Matrix protocol was followed for both the saliva samples and the crevicular fluid samples according to the manufacturer’s instructions.

Quantitative real-time PCR (qPCR) for HSV-1 was performed using the LightCycler^®^ 480 II system (Roche Diagnostics, Basel, Switzerland), as previously described (REF 1). The reaction mixture for each 20 µL reaction contained 10 µL LightCycler^®^ 480 Probes Master (2×) (Roche Diagnostics); 0.5 µM HSV-1 primer: GCAGTTTACGTACAACCACATACAGC (Biolegio, Nijmegen, The Netherlands); 0.5 µM HSV-2 primer: AGCTTGCGGGCCTCGTT (Biolegio, Nijmegen, The Netherlands); 0.2 µM HSV-1-specific TaqMan probe (FAM-labeled): CGGCCCAACATATCGTTGACATGGC (Biolegio, Nijmegen, The Netherlands); 2 µL of sample DNA and Nuclease-free water to a final volume of 20 µL. The thermal cycler conditions were as follows: Initial denaturation: 95 °C for 10 min; 45 cycles of denaturation at 95 °C for 10 s and annealing at 60 °C for 15 s. Fluorescence signals were detected in the FAM channel using the LightCycler^®^ 480 Software, and quantification cycle (Cq) values were determined automatically.

Quantitative real-time PCR (qPCR) for EBV detection was performed using the LightCycler^®^ 480 II system (Roche Diagnostics, Basel, Switzerland), as previously described (REF 2). Each 20 µL reaction contained the following components: 10 µL LightCycler^®^ 480 Probes Master (2×) (Roche Diagnostics); 0.5 µM forward primer (EBV-F): GGAACCTGGTCATCCTTTGC (Biolegio, Nijmegen, The Netherlands); 0.5 µM reverse primer (EBV-R): ACGTGCATGGACCGGTTAAT (Biolegio, Nijmegen, The Netherlands); 0.2 µM EBV-specific TaqMan probe (HEX-labeled): CGCAGGCACTCGTACTGCTCGCT (Biolegio, Nijmegen, The Netherlands); 2 µL of sample DNA; Nuclease-free water to a final volume of 20 µL. Thermal cycling was carried out under the following conditions: an initial denaturation at 95 °C for 10 min, followed by 45 cycles of denaturation at 95 °C for 10 s and annealing/extension at 60 °C for 15 s. Fluorescence was measured in the HEX detection channel using the LightCycler^®^ 480 software, and quantification cycle (Cq) values were determined automatically.

The amplification efficiency of the PCR assay was evaluated using serial 10-fold dilutions of purified HSV-1 DNA. The resulting Ct values ranged from 9.1 to 22.6, with a correlation coefficient (R^2^) of 0.998 and a slope of 3.40, indicating high amplification efficiency and assay reliability [44].

Bacterial identification was performed using the Paro x test panel (ADD Laboral). This panel employs a microbiological quantitative PCR (qPCR) method to detect bacterial DNA in clinical samples. It targets 11 specific periodontal pathogens, namely: *Aggregatibacter actinomycetemcomitans (Aa)*, *Porphyromonas gingivalis (Pg)*, *Tannerella forsythia (Tf)*, *Treponema denticola (Td)*, *Fusobacterium nucleatum (Fn)*, etc. The assay provided both qualitative (presence/absence) and quantitative results. Bacterial load was expressed as colony-forming units (CFU), determined digitally based on qPCR amplification data.

### 2.5. Statistical Analysis

Data analysis and interpretation were performed using IBM SPSS Statistics V31. SPSS, which stands for Statistical Package for the Social Sciences, is a widely used software suite for statistical analysis across various fields, including social sciences, healthcare, education, marketing, and the corporate sector. To ensure the accuracy and reliability of the results, they were additionally verified using two artificial intelligence tools: ChatGPT 4 (OpenAI) and Gemini 2.5-pro version(Google).

## 3. Results

### 3.1. Clinical and Demographic Profile of the Study Population

Demographic characteristics (age, gender, smoking status, periodontal disease) and implant function and disease status details are presented in Table 1. The implant location and disease status are described in Table 2.

The study included 43 patients. A total of 19 participants were diagnosed with periodontal disease, and 7 reported a history of smoking. The patients were on average 54.5 years old, 21 females, 22 males. A total of 100 implants were sampled, 74 with a healthy status, and 26 with peri-implantitis. They were in function for an average of 3.9 years. 32 implants had less than 5 years of functional loading time, and 68 had more than 5 years of functional loading time. A total of 44 implants were located in the maxila, and 56 in the mandible. A total of 50 teeth were sampled, of which 19 were diagnosed with periodontal disease and 31 exhibited a healthy periodontal status.

#### Element Type Distribution

The distribution of molars, premolars, and incisors across HI, PI, and TT samples is presented in Table 3.

The analysis confirms a balanced representation of molars (*n* = 66), premolars (*n* = 60), and incisors (*n* = 24) across various jaw regions and sampled element types. 

### 3.2. Probing Depth Analysis by Site and Inflammatory Markers (BoP, SUP)

The analysis of PPD for HI, PI, and TT is presented in Table 4.

The analysis revealed that the lingual (L) site exhibited the highest mean probing depth (3.75 mm), followed by the distal (D), buccal (B), and mesial (M) sites, as presented in Table 5, while the average is presented also in Table 5.

The majority of PPD values, ranging from 1 to 5 mm, were observed in the HI and TT groups. In contrast, PPD values between 5 and 8 mm were strongly associated with PI, being present in 17 out of 19 cases. Notably, no site classified as HI exhibited a PPD greater than 5 mm. TT cases generally remained within the clinically acceptable PPD range, with only a limited number of pathological values recorded.

PI sites demonstrated significantly greater PPD across all anatomical locations compared to HI sites. Lingual surfaces were particularly affected, with the deepest PPD recorded (8.00 mm), highlighting their critical role in peri-implant monitoring and the need for targeted hygiene protocols. 

The lingual site exhibited the highest BoP rate at 75%. Buccal and distal sites demonstrated moderate BoP rates of 38.57% and 32.73%, respectively, suggesting a relatively higher inflammatory load in these regions. In contrast, mesial sites showed the lowest BoP rate at 30%, as presented in Table 6.

The analysis in Table 6 reveals a significant variation in BoP rates across clinical categories. PI sites exhibited a markedly high BoP prevalence of 96.15%, reinforcing its strong association with active inflammation and ongoing tissue breakdown. In contrast, HI sites demonstrated BoP in 28.38% of cases, potentially indicating early or subclinical inflammatory changes. TT samples presented the lowest BoP rate at 18.00%, reflecting comparatively healthier periodontal conditions.

SUP was highly prevalent in PI cases, occurring in 96.15% of instances, whereas it was relatively rare in TT and HI sites, as shown in Table 7. Lingual sites exhibited the highest site-specific suppuration rate at 50%. Buccal and mesial sites showed similar suppuration rates (~20%), while distal sites demonstrated slightly lower levels (~16%), as summarized in Table 7.

### 3.3. Assessment of Viral Presence and Distribution

Viral DNA analysis in Table 8 revealed distinct detection patterns among the three herpesviruses examined. EBV was identified in 13 out of 43 patient samples (30.2%), while HSV was detected in 3 samples (7.0%). CMV was not detected in any of the specimens analyzed.

Only EBV exhibited a statistically moderate negative correlation with probing PPD (r = −0.48) in non-smoking individuals and those diagnosed with periodontal disease. This inverse relationship may suggest a tendency for EBV to localize in sites with less advanced tissue destruction, potentially reflecting differences in the local immune environment or microbial composition at shallower sites. No correlations could be established for CMV or HSV, as the lack of variation (i.e., constant negative values) precluded statistical analysis. 

EBV exhibited the highest mean levels on lingual surfaces, whereas herpes simplex virus showed peak detection on mesial and distal aspects, as presented in Table 9.

**Figure 1:**
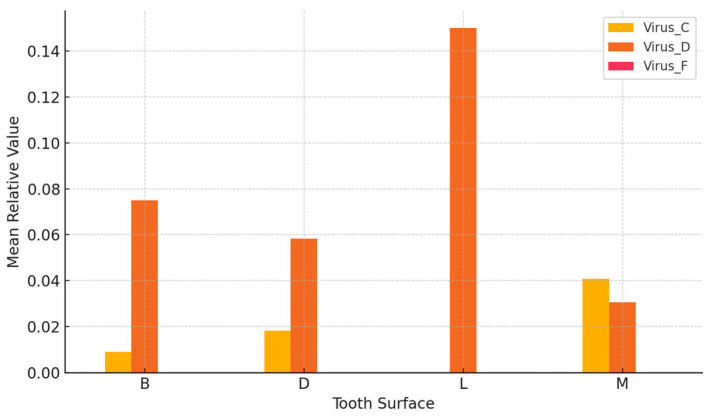
Mean viral levels by tooth surface location.

Moderate variation in viral load was observed in Figure 1 across different sampling sites, with lingual and mesial surfaces exhibiting relatively higher concentrations.

### 3.4. Association Between Systemic Antibody Responses and the Presence of Viral DNA in Oral Samples

**Figure 2:**
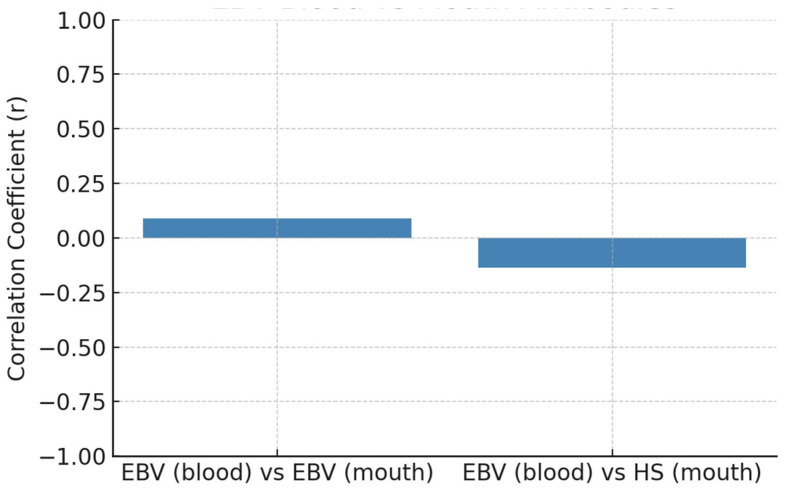
EBV systemic antibody titer vs. oral viral presence.

**Figure 3:**
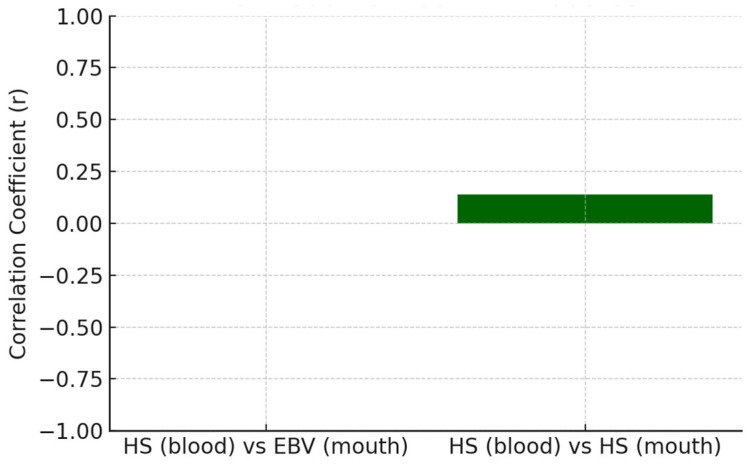
HS systemic antibodies titer vs. oral viral presence.

The relationship between systemic antibody responses and the presence of viral DNA in oral samples was evaluated to explore the host immune status in relation to local viral colonization in Table 10, being depicted for EBV in Figure 2 and for HS in Figure 3.

The correlations between systemic antibody levels and oral viral DNA presence were generally weak (r < 0.15), indicating minimal correspondence between these compartments. EBV and herpes, HSV demonstrated a slight positive association. However, these relationships were insufficiently robust to infer a reliable biological linkage. CMV could not be assessed due to uniform antibody values within the dataset.

### 3.5. Analysis of Systemic Antibody Profiles and Their Distribution Across Clinical Groups

The objective of this analysis was to assess whether systemic antibody levels against CMV, EBV, and HSV differ across distinct clinical contexts: HI, PI, and TT as presented in Table 11.

**Figure 4:**
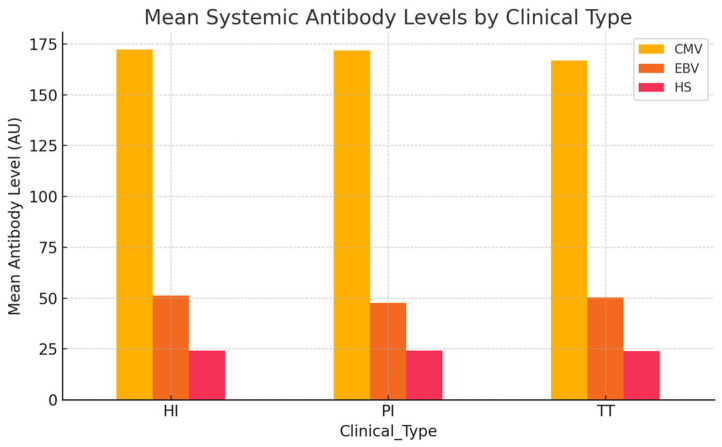
Mean systemic antibody levels by clinical type.

Systemic antibody levels were generally comparable across the clinical groups, as presented in Figure 4. HI subjects exhibited slightly higher mean antibody titers for CMV and EBV compared to individuals with PI and those with TT, a finding that is somewhat unexpected given the inflammatory profile of PI. Nevertheless, these differences were minimal and did not reach statistical significance. Overall, systemic antibody levels against CMV, EBV, and HSV showed no clear variation among the clinical categories. 

Our analysis investigated whether systemic antibody levels against CMV, EBV, and HSV differ according to individuals’ smoking status and periodontal disease presence.

Table 12 and Table 13 summarize the mean antibody levels by group.

**Figure 5:**
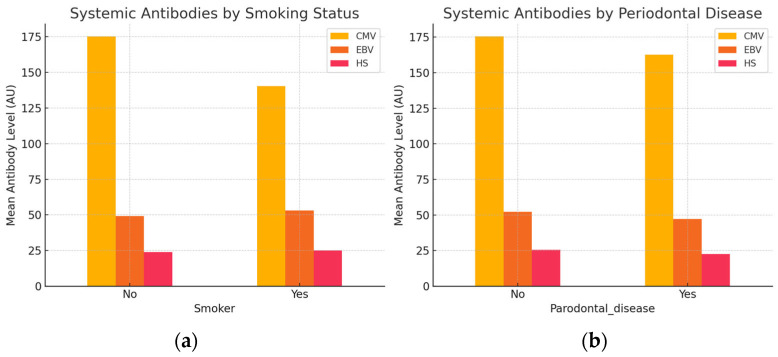
Analysis of systemic antibody levels by group (**a**) smoker status and (**b**) periodontal disease.

Smokers exhibited lower systemic antibody levels against CMV but slightly elevated antibody titers for EBV and HSV compared to non-smokers, as presented in Figure 5a. Conversely, in Figure 5b, individuals with periodontal disease demonstrated reduced antibody levels across all three viruses. Overall, systemic antibody responses appear to vary modestly in relation to smoking behavior and periodontal disease presence.

We also explored the potential correlation between systemic immune responses and the composition of the oral bacterial microbiota. By examining these relationships, we aim to gain insight into whether systemic antiviral immunity may play a role in shaping local microbial communities within the oral environment. 

**Figure 6:**
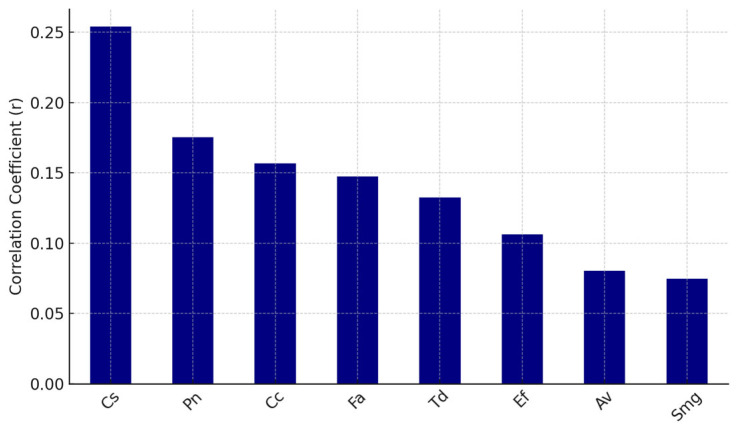
Top positive correlations: EBV (systemic) vs. bacteria.

**Figure 7:**
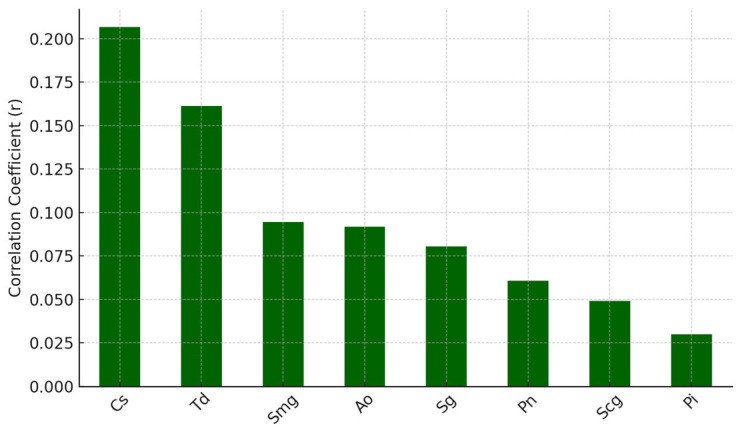
Top positive correlations: HS (systemic) vs. bacteria.

Pearson correlation coefficients were calculated between each virus and each bacterial species. The top eight bacteria positively correlated with EBV and HS antibodies are shown in Figure 6 and Figure 7, respectively.

Systemic antibody levels against EBV and HSV demonstrated modest positive correlations with the presence of specific oral bacterial taxa, including *Campylobacter rectus* (Cr), *Fusobacterium alocis* (Fa), *Prevotella melaninogenica* (Pm), and *Treponema forsythia* (Tf). 

## 4. Discussion

The interpretation of our findings must be considered in light of the limited literature available on the association between herpesviruses and PI [45,46]. Due to the limited number of studies published on this subject, the findings of the present investigation could be directly compared with only five prior reports [33,37,44,47,48].

Among the studies reviewed, both Kato et al. [47] and Marques Filho et al. [48] employed case–control designs, comparing patients with peri-implantitis to periodontally healthy individuals. In contrast, Jankovic et al. [33] conducted genotypic analysis exclusively on peri-implantitis sites, without including healthy controls. Canullo et al. [44] explored the modulatory role of EBV in peri-implant pathology rather than its diagnostic utility. Notably, Verdugo et al. [37] adopted a split-mouth design.

EBV was a consistent focus across all five studies reviewed. Notably, Jankovic et al. [33] were the only group to investigate’ cytomegalovirus (CMV/HCMV) genotypes, while Marques Filho et al. [48] assessed a broader range of human herpesviruses but used only saliva samples. Kato et al. [47] uniquely integrated both viral and bacterial targets, specifically examining the co-detection of EBV and *Porphyromonas gingivalis*. All studies employed PCR-based detection methods.

Limitations were evident across studies, including relatively small sample sizes, 30–42 patients, that constrain statistical power and generalizability. Variability in CMV detection may also reflect regional or genotype-specific differences. Furthermore, bacterial co-infection was not consistently assessed, as observed in the study by Jankovic et al. [33], limiting the ability to fully evaluate microbial–viral interactions. Importantly, Canullo et al. [44] ’s conclusion that EBV may act more as a biological enhancer than a standalone pathogenic marker is aligned with growing evidence supporting a synergistic role of viral and bacterial agents in PI.

Consistent with the present findings, these studies have underscored the potential involvement of EBV in peri-implant inflammatory processes. Kato et al. [47] reported the concurrent detection of EBV DNA and *Porphyromonas gingivalis* in peri-implant crevicular fluid in the Japanese population. This observation aligns with our own data, which revealed higher EBV levels at lingual and mesial implant surfaces and positive associations with certain bacterial taxa, including *Campylobacter rectus*, *Fusobacterium alocis*, *Prevotella melaninogenica*, and *Treponema forsythia*. 

Jankovic et al. [33] identified associations between specific EBV and CMV genotypes and peri-implant inflammation; however, CMV was not detected in our cohort. Similarly, Canullo et al. [44] reported a significantly greater prevalence of EBV in PI relative to HI, reflecting the pattern observed in our study, where EBV was detected in 30.2% of patients—most notably among non-smokers with concomitant periodontal disease. Marques Filho et al. [48] also documented elevated EBV and HSV levels in saliva samples from patients with PI, which contrasts with the lower HSV detection rate (7.0%) and absence of CMV in our data. Verdugo et al. [37] also demonstrated a higher prevalence of EBV in diseased PI. 

While these studies differ in design, population, and detection methods, they collectively support the potential role of EBV in inflammatory processes around implants, while the role of CMV and HSV appears to be less consistently established across studies.

The moderate variation in EBV presence by site—especially the increased presence on lingual and mesial surfaces—suggests that anatomical niches may support viral persistence or reactivation, potentially due to differences in microbial colonization, plaque retention, or tissue immune properties.

The observation of weak correlations between systemic antibody levels and DNA viral load in oral samples supports the theory that systemic immunity reflects historical exposure, whereas oral viral presence may indicate localized reactivation. The study’s strengths and weaknesses are discussed below.

The present study employed a multisite sampling strategy, including blood, saliva, and peri-implant crevicular fluid. To the best of our knowledge, this is the first study to analyze all three of them.

No previous study has included samples collected from the adjacent tooth of either healthy implants or implants affected by PI.

Unlike the study by Jankovic et al. [33], a well-defined control group comprising healthy implant (HI) and teeth (TT) sites was included, allowing for more robust comparative analysis. Moreover, a clear clinical association was observed between EBV positivity and patient-related factors such as smoking status and a history of periodontal disease. Additionally, the study provided a detailed assessment of site topography (buccal, lingual, mesial, and distal surfaces), an anatomical dimension that has not been explored in previous investigations.

The sampling methodology in this study relied on sterile paper points for subgingival collection. However, subsequent evaluations have indicated that paper points may carry exogenous DNA, potentially introducing non-oral microbial signatures into DNA-based analyses. To minimize this risk in future analyses, sterile curettes are recommended for microbial sampling in molecular studies [49].

Despite the relatively larger sample size in the present study compared to comparable investigations, uneven distribution of variables such as smoking status and implants diagnosed with peri-implantitis among participants poses challenges to the validity of direct comparisons.

## 5. Conclusions

EBV was present in about one-third of peri-implantitis patients, particularly at lingual/mesial surfaces, in non-smokers and individuals with periodontal disease, in less severe lesions. 

Systemic antibody levels for CMV, EBV, and HSV were generally comparable across clinical categories HI, PI, and TT. This suggests that local peri-implant inflammatory status does not substantially influence systemic viral antibody responses.

EBV and HSV antibody levels showed modest positive associations with certain bacterial taxa, including *Campylobacter rectus*, *Fusobacterium alocis*, *Prevotella melaninogenica*, and *Treponema forsythia*.

## Figures and Tables

**Table 1:** Demographic and implant function summary.

Characteristic	Value
Average Age (years)	54.5
Gender (F/M)	21 (49%)/22 (51%)
Smoking Status (Non-smoker/Smoker)	36 (84%)/7 (16%)
Periodontal Disease (No/Yes)	24 (56%)/19 (44%)
Implant Function ≤ 5 years (HI/PI)	32/5
Implant Function > 5 years (HI/PI)	42/21
Total Implants ≤ 5 years (% PI)	37 (13.5%)
Total Implants > 5 years (% PI)	63 (33.3%)

**Table 2:** Implant location and disease status summary.

Jaw Region	Count
Maxilla Implants	44
Mandible Implants	56
HI	74
TT	50
PI	26

**Table 3:** Element type distribution table.

Element Type	Jaw	Incisor	Molar	Premolar
HI	Mandible	5	30	8
HI	Maxilla	5	11	15
PI	Mandible	1	8	4
PI	Maxilla	2	6	5
TT	Mandible	4	5	21
TT	Maxilla	7	6	7
HI	Total	10	41	23
PI	Total	3	14	9
TT	Total	11	11	28

**Table 4:** PPD interval frequencies by clinical site status.

PPD Interval	HI	PI	TT
1–5 mm	74	9	48
5–8 mm	0	17	2

**Table 5:** Mean and group-specific PPD by site.

Site	Mean PPD (mm)	HI (mm)	PI (mm)
B	2.93	2.21	6.0
D	2.98	2.39	5.62
L	3.75	2.0	8.0
M	2.85	2.36	6.0

**Table 6:** Bleeding on probing (BoP) by site and sample type.

Category	Total Cases	Positive BoP	BoP Rate (%)
B	70	27	38.57
D	55	18	32.73
L	4	3	75.00
M	20	6	30.00
HI	74	21	28.38
PI	26	25	96.15
TT	50	9	18.0

**Table 7:** Suppuration rates by sample type and anatomical site.

Category	Total Cases	Positive Suppuration	Suppuration Rate (%)
HI	74	2	2.70
PI	26	25	96.15
TT	50	4	8.00
B	70	15	21.43
D	55	9	16.36
L	4	2	50.00
M	20	4	20.00

**Table 8:** Viral presence and distribution.

Virus	Smoker_No	Smoker_Yes	Periodontal_Yes	Periodontal_No
PPD	1.00		1.00	
HS				
EBV	−0.48		−0.48	
CMV				

**Table 9:** Tooth surface presence of viruses.

Tooth Surface	Virus EBV(D)	Virus_HS(C)	Virus_CMV(F)
B	0.009	0.075	0.000
D	0.018	0.058	0.000
L	0.000	0.150	0.000
M	0.041	0.031	0.000

**Table 10:** Analysis of systemic antibody titer vs. oral samples.

Blood vs. Oral	CMV_oral	EBV_oral	HS_oral
CMV_blood		0.06	0.08
EBV_blood		0.09	−0.14
HS_blood		−0.00	0.14

**Table 11:** Analysis of systemic viral profile.

Virus	HI	PI	TT
CMV	172.29	171.72	166.77
EBV	51.24	47.57	50.31
HS	24.18	24.10	23.95

**Table 12:** Analysis of antibodies for smoker status groups.

Virus	Non-Smoker	Smoker
CMV	175.31	140.33
EBV	49.29	53.19
HS	23.99	24.97

**Table 13:** Analysis of antibodies for periodontal groups.

Virus	No Periodontal Disease	With Periodontal Disease
CMV	175.60	162.73
EBV	52.29	47.20
HS	25.47	22.62

## Data Availability

The original contributions presented in this study are included in the article. Further inquiries can be directed to the corresponding author.
